# Intra-operative red blood cell transfusion and mortality after cardiac surgery

**DOI:** 10.1186/s12871-019-0738-2

**Published:** 2019-05-04

**Authors:** Eline A. Vlot, Lisa Verwijmeren, Ewoudt M. W. van de Garde, Geoffrey T. L. Kloppenburg, Eric P. A. van Dongen, Peter G. Noordzij

**Affiliations:** 1 Department of Anesthesiology, Intensive Care and Pain Medicine, St Antonius Hospital, Koekoekslaan 1, 3430 EM Nieuwegein, The Netherlands; 2 Department of Clinical Pharmacy, St Antonius Hospital, Nieuwegein, The Netherlands; 3 Department of Cardiac Surgery, St Antonius Hospital, Nieuwegein, The Netherlands

**Keywords:** Cardiac surgery, Blood transfusion, Postoperative mortality

## Abstract

**Background:**

Anemia in cardiac surgery patients has been associated with poor outcomes. Transfusion of red blood cells during surgery is common practice for perioperative anemia, but may come with risks. Little is known about the association between intra-operative transfusion and mortality in patients undergoing cardiac surgery.

**Methods:**

Single centre historical cohort study in 2933 adult patients undergoing coronary surgery with or without aortic valve replacement from June 2011 until September 2014. To estimate the odds ratio for mortality in patients receiving intra-operative transfusion, a propensity score based logistic regression analysis was performed.

**Results:**

Intra-operative transfusion was associated with a more than three-fold increased risk of 30-day mortality. Patients in the highest quartile of probability of transfusion were older (age 75 vs 66; *P* <  0.001), had a higher EuroSCORE (6 vs 3; *P* <  0.001), had lower preoperative hemoglobin levels (7.6 vs 8.9 mmol/l; *P* < 0.001), had combined surgery more often (CABG + AVR in 33.4% of cases vs 6.6% (*P* < 0.001) and a longer duration of surgery (224 vs 188 min; *P* < 0.001). The association between intra-operative transfusion and mortality persisted after adjustment for these risk factors (adjusted OR 2.6; *P* = 0.007).

**Conclusions:**

Intra-operative transfusion of red blood cells was found to be associated with increased mortality in adults undergoing coronary surgery. Preoperative patient optimization may improve perioperative outcomes by reducing the likelihood of requiring transfusion and thus its associated risk.

## Background

Blood loss reduces the oxygen delivery capacity due to hypovolemia and anemia. Healthy patients are able to increase tissue oxygenation by a physiologic increase in cardiac output and enhanced oxygen extraction from erythrocytes. In patients with coronary disease these compensatory mechanisms are limited and without treatment anemia may lead to myocardial ischemia and subsequent myocardial infarction. Anemia in cardiac surgery patients is common and has been associated with poor outcome [1–3]. During surgery, ongoing blood loss and hemodilution as a result of cardiopulmonary bypass (CPB) causes low hemoglobin (Hb) levels. Transfusion of red blood cells (RBC) is the current standard practice for perioperative anemia, but RBC transfusion is not without risks. Historical cohort studies that evaluated the influence of RBC transfusion in patients with perioperative anemia have reported an increased risk for adverse events, including mortality [4–8]. Additionally, the optimal transfusion trigger in cardiac surgery patients is largely unknown [9, 10]. Especially during surgery, patients may temporarily tolerate lower Hb levels as metabolism is low and oxygen needs are reduced due to hypothermia and anesthesia. Further insight in the risk of intraoperative RBC transfusion to treat anemia may aid cardiac anesthetists to further improve patient blood management. This study aimed to investigate the association between intraoperative RBC transfusion and mortality in patients undergoing coronary surgery.

## Methods

### Study design and population

All patients who underwent isolated coronary artery bypass graft surgery (CABG) or combined CABG and aortic valve replacement (AVR) from June 2011 until September 2014 in St. Antonius Hospital, Nieuwegein, The Netherlands, were eligible for inclusion in this historical cohort study. Local Medical Research Ethics Committee approval was obtained with a waiver for patient informed consent (MEC-U; Research and Development Department St. Antonius Hospital, trial number V15.020).

### Data collection and potential confounding factors

Data regarding medical history and preoperative drug therapy were registered at the outpatient anesthesia clinic during routine preoperative screening. Standard laboratory hematology and coagulation parameters were retrieved from the hospital electronic patient records. Information regarding surgery, CPB, transfusion products and postoperative blood loss were collected from computerized perioperative medical records (Metavision Suite 5.46.44, iMDsoft®, Düsseldorf, Germany).

Prior to analysis, variables that were potentially associated with RBC transfusion were specified based on previous literature reports and biological plausibility including: age, sex, body mass index (BMI), preoperative Hb concentration, preoperative creatinine concentration, smoking, chronic obstructive pulmonary disease (COPD), hypertension, diabetes, peripheral artery disease, previous stroke, left ventricular ejection fraction (LVEF), previous cardiac surgery, unstable angina, emergency surgery, duration of operation time, autologous red blood cell transfusion, use of an internal mammary artery and type of surgery [11, 12].

### Outcome measures

The primary outcome measure was postoperative mortality within 30 days after surgery, further denominated as mortality. Registration of mortality was conducted in the context of a national registry of cardiac interventions in The Netherlands (Netherlands Heart Registry) [13].

### Clinical management

#### Anesthesia management and cardiopulmonary bypass

During the study period antiplatelet therapy (APT) was routinely discontinued 5 to 10 days prior to an elective surgical procedure. Routine perioperative anesthesia care included induction of anesthesia with midazolam, propofol, fentanyl and pancuronium and maintenance of anesthesia with propofol and fentanyl or remifentanyl. Vasoactive medications e.g. norepinephrine, dopamine, enoximone and nitroglycerine were used by discretion of the attending anesthetist. All patients received antimicrobial prophylaxis (cefazolin) at induction of anesthesia followed by additional cefazolin every 4 h for the duration of surgery and for 48 h in case of valve replacement. Intraoperative TEE as part of hemodynamic monitoring was routinely performed during valve surgery. In isolated CABG, use of TEE monitoring was left to the discretion of the attending anesthetist.

CPB consisted of a miniaturized extracorporeal circulation (MECC) or a conventional extracorporeal circulation (ECC) according to the preference of the surgeon and type of procedure. For CPB, non-pulsatile perfusion was used with a flow of 2.0 to 2.4 L/min/m^2^. All patients received heparin before CPB to achieve an adequate kaolin activated clotting time (ACT > 300 s for MECC and > 400 s for ECC). Additional heparin was administered when needed to keep ACT on target. After aortic cross-clamping, cardiac arrest was initiated using a cold crystalloid or blood (in case of MECC) cardioplegia solution. During CPB patients were cooled to a rectal temperature of 32 °C to 34 °C. Patients were weaned from CPB after rectal temperature reached 35.5 °C. In general, intraoperative management targeted a SvO_2_ of 65% and a MAP of 50 mmHg during CPB. According to institutional recommendations, heparin was reversed with protamine sulphate, 1 mg for every 100 U of previously administered heparin.

After surgery patients were transferred to the ICU and weaned from mechanical ventilation after exhibiting complete recovery from anesthesia, hemodynamically stability with no evidence of significant bleeding, core temperature > 36 °C and normal blood gas values. Patients were discharged from the ICU the following morning after meeting institutional discharge criteria.

#### Blood transfusion management

Blood product transfusion was performed according to a local transfusion protocol. The trigger for intraoperative RBC transfusion was a hematocrit < 20% during CPB or < 25% after separation from CPB. After surgery, RBC transfusion was at the discretion of the attending ICU physician and based on postoperative hemodynamics and chest tube output. A postoperative Hb value of 4.5 mmol/l (7.3 g/dL) was an absolute trigger for RBC transfusion.

Intraoperative plasma transfusion was based on the amount of blood loss; i.e. the number of transfused cell saver units or clinical signs of coagulopathy after protamine administration. Platelet transfusion depended on clinical signs of coagulopathy in combination with low platelet count (< 100 × 10^9^/l) or continuation of APT during surgery. The final decision for blood product transfusion was at the discretion of the attending physician.

Administration of factor concentrates was not part of routine blood management. Point-of-care hemostatic monitoring was implemented in Q2 of 2015 as a part of routine blood management. Intraoperative tranexamic acid use was left to the discretion of the attending anesthetist. Intraoperative cell salvage was routinely used.

### Statistical analysis

Categorical data are stated as number and percentages. Chi-square test or Fisher’s exact test were used to compare dichotomous variables between groups. Continuous data are described as median and interquartile range (IQR) as all continuous variables were non-normally distributed. The P-P plot was used to check for normality in distribution. Mann-Whitney U test or Student’s t-test was used to compare independent continuous variables between groups. To estimate the odds ratio for mortality in patients receiving intraoperative RBC transfusion a logistic regression analysis was performed using a propensity score (PS) as single confounder [14]. To define high-risk subgroups patients were stratified into four groups of equal size (quartiles) based on the sample distribution of the PS. Differences among quartiles were analysed using Mann-Whitney U test or Student’s t-test. A subgroup analysis was performed in the group of patients at highest risk of RBC transfusion (Q4) using multivariate regression analysis. A *p* value < 0.05 was considered statistically significant. For statistical analysis IBM SPSS version 22 (IBM Corp. Released 2013, SPSS Statistics for Windows, Version 22.0. Armonk, NY: IBM Corp.) was used.

#### Propensity score

The PS was constructed beforehand and represented the likelihood of receiving RBC transfusion based on pre- and intraoperative characteristics. Variables that were potentially associated with RBC transfusion were included in a multivariable logistic regression analysis and included age, sex, BMI, smoking, COPD, hypertension, diabetes, peripheral artery disease, previous stroke, LVEF, previous cardiac surgery, unstable angina, emergency surgery, additive EuroSCORE, preoperative Hb concentration, duration of operation time, autologous RBC transfusion, use of an internal mammary artery and type of surgery.

## Results

### Study population and baseline characteristics

A total of 2933 patients were included in the study. Mean age was 67 years (± 10) and 78% of patients were male. Most common comorbidities were hypertension (61%), diabetes mellitus (25%) and peripheral artery disease (12%). Median EuroSCORE was four [2–6] and 729 (25%) patients had a reduced LVEF (≤ 50%) prior to surgery. A majority of patients (87%) underwent isolated CABG surgery, IMA was used for grafting in 91% of cases and median duration of operation was 195 min [165–233]. In 209 patients (7%) an emergency procedure was performed. After surgery postoperative 12 h drain production was 580 ml [440–820] and postoperative Hb was 6.0 mmol/l [5.4–6.7] (9.7 g/dL [8.7–10.8]). In 3% of patients drain production exceeded 2000 ml/12 h. The median ICU length of stay was 21 h [18–43] and 123 (5%) patients had a re-sternotomy.

### Intra-operative transfusions

A total of 647 (22%) patients received a median of 3 [2–4] units of any intraoperative allogeneic transfusion. Plasma transfusion occurred in 210 patients (7%) with a median of 2 [2] units and platelet transfusion was performed in 567 patients (19%) with a median of 1 [1, 2] units. A total of 967 intraoperative RBC units were given to 488 patients (17%). Median number of transfused RBC units was 2 [1, 2]. Two-hundred-ninety-three patients (10%) received 2 or more RBC units. In patients with RBC transfusion, median Hb concentration at the end of surgery was 5.6 mmol/l [5.1–6.1] (9.0 g/dL [8.2–9.3]) versus 6.1 mmol/l [5.5–6.8] (9.3 g/dL [8.9–11.0]) in patients without RBC transfusion (*P* < 0.001). Table 1 shows differences in baseline characteristics of study patients according to intra-operative RBC transfusion before and after PS adjustment.

**Table 1:** Baseline characteristics according to intraoperative RBC transfusion

	No RBC transfusion	RBC transfusion	Unadjusted	PS adjusted
	(*n* = 2445)	(*n* = 488)	*p*-value	*p*-value
Patient characteristics				
Male gender, n (%)	2026 (82.9)	249 (51)	< 0.001	0.403
Age, yr	66 (60–73)	74 (68–79)	< 0.001	0.296
BMI, kg/m2	27 (25–30)	26 (24–29)	0.001	0.684
Smoking, n (%)	756 (30.9)	116 (23.8)	0.002	0.795
Chronic obstructive pulmonary disease, n (%)	279 (11.4)	66 (13.5)	0.365	0.984
Hypertension, n (%)	1466 (60.0)	321 (65.8)	0.016	0.688
Diabetes, n (%)	567 (23.2)	166 (34.0)	< 0.001	0.617
Peripheral artery disease, n (%)	252 (10.3)	90 (18.4)	< 0.001	0.688
Previous stroke, n(%)	161 (6.6)	50 (10.2)	0.004	0.708
Previous cardiac surgery, n (%)	60 (2.5)	33 (6.8)	< 0.001	0.894
Reduced LVEF (< 50%), n(%)	572 (23.4)	157 (32.2)	< 0.001	0.835
Unstable angina, n (%)	155 (6.3)	72 (14.8)	< 0.001	0.858
Emergency procedure, n (%)	156 (6.4)	53 (10.9)	0.001	0.948
EuroSCORE	3 (2–5)	6 (4–8)	< 0.001	0.290
Laboratory				
Hb, mmol/l	8.8 (8.2–9.3)	7.6 (7.0–8.4)	< 0.001	0.029
Creatinine, μmol/l	83 [73–96]	85 [71–109]	< 0.001	0.911
Procedural characteristics				
Duration of procedure, min	190 [161–227]	223 [186–265]	< 0.001	0.652
Use of IMA, n (%)	2251 (92.1)	412 (84.4)	< 0.001	0.927
Salvaged red cell volume reinfused (units)	1.8 [1.0–2.0]	2.0 [1.0–3.0]	< 0.001	0.809
CABG, n (%)	2225 (91.0)	330 (67.6)	< 0.001	0.875
CABG + AVR, n (%)	220 (9.0)	158 (32.4)	< 0.001	0.875

Data are presented as median (IQR), or frequencies (n, (%))

*PS* propensity score, *RBC* red blood cell, *BMI* body mass index, *LVEF* left ventricular ejection fraction, *Hb* hemoglobin, *IMA* internal mammary artery

### Propensity model

The adjustment for PS adequately balanced the groups for the baseline variables with exception of preoperative Hb (Table 1). There was some overlap for PS distributions between groups (0.5 ± 0.30 (range 0.0–1.0) for patients with RBC transfusion vs. 0.1 ± 0.15 (range 0.0–0.93) for patients without RBC transfusion; *P* < 0.001).

### Intra-operative RBC transfusion and postoperative mortality

Sixty patients (2%) died within 30 days after surgery. Sixty-two percent of patients who died had intra-operative RBC transfusion compared to 16% of survivors (*P* < 0.001). After propensity score adjustment, intraoperative RBC transfusion was associated with a more than three-fold mortality risk (OR 3.1; 95% CI 1.5–6.7; *P* = 0.003).

### Subgroup analysis in patients with high probability of transfusion

Table 2 presents differences in risk factors for mortality after stratification into quartiles according to PS. Patients with the highest probability of transfusion (Q4) were older (age 75 vs 66 for Q1-Q3; *P* < 0.001), had a higher EuroSCORE (6 vs 3 for Q1-Q3; *P* < 0.001), had lower preoperative hemoglobin levels (7.6 mmol/l (12.3 g/dl) vs 8.9 mmol/l (14.3 g/dl) for Q1-Q3; *P* < 0.001), had combined surgery more often (CABG + AVR in 33.4% of cases vs 6.6% in Q1-Q3 (*P* < 0.001) and a longer duration of surgery (224 vs 188 min for Q1-Q3; *P* < 0.001). Mortality was highest in Q4 (5.1% compared to 0.4, 0.7 and 2.2% in Q1–3 respectively). In patients with the highest probability of transfusion intraoperative RBC transfusion was associated with a 4.1-fold risk of mortality (PS adjusted OR 4.1 and 95% CI 1.3–12.6, *P* = 0.016). In Q4, OR for RBC associated mortality changed with 7% after adding plasma transfusion to the PS adjusted mortality (OR 3.8, *P* = 0.021). After adjustment for differences in age, EuroSCORE, preoperative Hb, type and duration of surgery, the OR for mortality in patients with intraoperative RBC transfusion in Q4 was 2.6 (95% CI 1.3–5.1, *P* = 0.007).

**Table 2:** Differences in patient characteristics, RBC transfusion and mortality according to propensity score subgroups

	Q1 (*n* = 683)		Q2 (*n* = 684)		Q3 (*n* = 684)		Q4 (*n* = 683)	
	RBC - (*n* = 672)	RBC + (*n* = 11)	RBC - (*n* = 665)	RBC + (*n* = 19)	RBC - (*n* = 612)	RBC + (*n* = 72)	RBC - (*n* = 360)	RBC + (*n* = 323)
Age, y	63 [56–68]	61 [57–65]	66 [59–71]	68 [64–77]	70 [63–75]	69 [63–76]	74 [68–79]	75 [71–80]
EuroSCORE	2 [1–4]	2 [1–2]	3 [1–4]	4 [2–6]	4 [3–6]	4 [3–6]	6 [5–8]	7 [5–9] **
Hemoglobin, mmol/l	9.5 [9.1–9.8]	9.6 [9.3–9.8]	8.8 [8.5–9.2]	8.9 [8.4–9.1]	8.4 [8.0–8.8]	8.5 [8.0–8.8]	7.8 [7.2–8.3]	7.4 [6.8–7.9] **
CABG + AVR, n (%)	9 (1)	0	36 (5)	0	78 (13)	13 (18)	89 (28)	139 (39)**
Duration procedure, min	172 [148–199]	142 [115–230]	197 [169–226]	189 [178–225]	200 [169–236]	206 [177–235]	214 [180–252]	229 [195–275] **
Mortality, n (%)	3 (0.4)	0	5 (0.8)	0	11 (1.8)	4 (5.6) *	4 (1.1)	31 (9.6)**

Data are presented as median [IQR] or frequencies (n, (%)). Q = quartile. Hemoglobin = preoperative value

Within stratum baseline covariate comparison between RBC transfused and not transfused patients was significant in the highest quartile * = *P* < 0.05, ** = *P* < 0.01

## Discussion

This historical cohort study in patients undergoing coronary surgery showed that intraoperative RBC transfusion was associated with a more than three-fold increased risk for mortality. The association between intraoperative transfusion and mortality remained present in a subgroup of patients with high probability of RBC transfusion after adjustment for multiple risk factors.

Anemia predisposes to myocardial ischemia in patients with coronary disease, especially during cardiac surgery when ongoing blood loss and hemodilution lower the oxygen carrying capacity of blood. Intraoperative RBC transfusion is a common but potential harmful treatment of anemia, which emphasizes the paradox that both anemia and transfusion are associated with adverse outcome in cardiac surgery patients [1–8]. This raises the question to what extend anemia should be accepted during coronary surgery and when RBC transfusion becomes ineluctable. The recent TRICS III study has provided further insight in RBC transfusion thresholds in cardiac surgery patients. In a multicenter trial patients undergoing on-pump cardiac surgery were randomized to a restrictive (< 7.5 g/dL) or liberal (< 9.5 g/dL) RBC transfusion strategy, in the operating room and intensive care unit. After 6 months follow up a perioperative restrictive transfusion strategy was non-inferior to a liberal strategy in terms of a composite endpoint including death, myocardial infarction, stroke or new onset renal failure [15]. In our cohort, the median Hb concentration at the end of surgery in patients with RBC transfusion was 5.6 mmol/l (9.0 g/dL) and substantially higher than the restrictive transfusion threshold in TRICS III. These results suggest that increased mortality rates in our study population are more likely to be associated with RBC transfusion than intraoperative anemia. In addition, when considering the general rule of thumb that a single unit of red cells results in an Hb increment of 1 g/dL, transfusion of a single RBC unit could have been withheld in 348 patients [16].

Despite transfusion guidelines in cardiac surgery patient blood management varies between physicians. Especially in surgical patients at older age or with more extensive comorbidity a tendency exists towards a more liberal transfusion strategy [17–19]. This was confirmed by the results of our study, patients with the highest probability of RBC transfusion were characterized by older age, a higher EuroSCORE and more complex surgery. These findings illustrate that besides Hb concentration anesthetists and surgeons consider multiple clinical factors in their treatment decision for intraoperative transfusion. However, also in more vulnerable patients RBC transfusion has been associated with increased risk for adverse outcome. In the TITRE2 study a liberal transfusion strategy (Hb > 9.0 g/dL) was compared to a restrictive transfusion strategy (Hb > 7,5 g/dL) in a multi-centre, randomized, controlled trial in adult patients after cardiac surgery [9]. Secondary analyses that studied the effect of transfusion showed that patients with older age, higher logistic EuroSCOREs and longer duration of surgery were more likely to be treated with RBC transfusion [19]. Mortality in patients with RBC transfusion was higher (3.7%) compared to non-transfused participants (2.7%) [19]. The recent TRICS III trial also studied the impact of a liberal versus restrictive transfusion strategy in cardiac surgery patients, but had a different design [15]. Patients were randomized prior to surgery and intra-operative RBC transfusion management was part of the study protocol. In that study, a sensitivity analysis of patients 75 years or older showed that a restrictive transfusion strategy appeared to be superior to a liberal strategy with regard to the composite endpoint of death, myocardial infarction and new renal failure requiring dialysis (OR 0.77 and 95% CI 0.62–0.96, *P* = 0.001) [15]. Although both TITRE2 and TRICS III have not primarily focused on the optimal transfusion trigger in the older cardiac surgery population, the results of subgroup analyses demonstrate that especially in older more frail patients intra-operative RBC transfusion seems to be associated with increased risk for adverse outcome.

Although coronary surgery patients are potentially more vulnerable to anemia due to fixed coronary stenosis, a restrictive RBC transfusion strategy in patients with stable cardiovascular disease is now considered relatively safe and has been recommended in recent literature reports [20–24]. It seems to become increasingly clear that myocardial revascularization primarily obviates the ischemic complications and that intraoperative anemia (Hb > 7.5 g/dL) plays no major role in adverse outcome. These findings may justify a restrictive transfusion strategy, especially in the operating room, where anemia is often the result of hemodilution and at least partially reversible. Additionally, during surgery the oxygen needs of tissues are reduced due to anesthesia and hypothermia. To further reduce the risk of intraoperative RBC transfusion clinicians should focus on diagnosing and treating preoperative anemia, especially in older patients and/or those with multiple comorbidities.

This analysis has some limitations, although our historical cohort study resembles real-world practice the results may have been influenced by confounding factors. Instead of random transfusion allocation based on a predefined transfusion trigger (as in randomized trials), RBC transfusion decision were based on a combination of a local transfusion protocol and individual physician preferences. Therefore, to assess the outcome between patients with and without intraoperative RBC transfusion propensity score analysis was used to account for systematic differences in baseline characteristics between patients with and without transfusion. However unmeasured confounders may have influenced the association between RBC transfusion and mortality. This study was a retrospective observational investigation before point-of-care driven patient blood management was standard practice in our operating theatre and covers a period in which the available evidence regarding restrictive RBC transfusion strategy was limited. Since then it has become increasingly clear that reducing transfusion rates with evidence-based patient blood management programs can provide significant clinical and economic advantages. At last, this study reports a short term negative association with intraoperative RBC transfusion while transfusion may have long-term consequences.

## Conclusion

Intra-operative transfusion of red blood cells was found to be associated with increased mortality in adults undergoing coronary surgery. Future work should focus on reducing the probability of intraoperative RBC transfusion by preoperative patient optimization and treatment of preoperative anemia.

## Abbreviations

ACT: Kaolin activated clotting time
APT: Anti-platelet therapy
AVR: Aortic valve replacement surgery
BMI: Body mass index
CABG: Coronary artery bypass grafts surgery
COPD: Chronic obstructive pulmonary disease
CPB: Cardio-pulmonary bypass
ECC: Conventional extracorporeal circulation
Hb: Hemoglobin level
ICU: Intensive care unit
IMA: Internal mammary artery
IQR: Interquartile range
LVEF: Left ventricular ejection fraction
MECC: Miniaturized extracorporeal circulation
OR: Odds ratio
PS: Propensity score
Q: Quartile
RBC: Red blood cell transfusion
